# The unintended consequences of school-based health and nutrition policies: a systematic review

**DOI:** 10.3389/fpsyg.2024.1356663

**Published:** 2024-07-05

**Authors:** Samantha L. Turner, Alexis M. Libert, Grace Haase, Zhaoyi Pan, Andrew Austin, C. Alix Timko

**Affiliations:** ^1^Department of Child and Adolescent Psychiatry and Behavioral Sciences, Children’s Hospital of Philadelphia, Philadelphia, PA, United States; ^2^Department of Psychiatry, Perelman School of Medicine, University of Pennsylvania, Philadelphia, PA, United States

**Keywords:** unintended consequences, health policies, school-based policies, unhealthy weight control behavior, body mass index, obesity prevention

## Abstract

**Background:**

Significant funding and attention are directed toward school-based health and nutrition interventions. Less attention is given to the potential unintended consequences of these policies, especially those that target children and adolescents. This systematic review aimed to elucidate the unintended consequences of school-based health and nutrition policies in the United States.

**Methods:**

We conducted a systematic review, adhering to PRISMA guidelines, to analyze quantitative, qualitative, and mixed methods research conducted between January 2013 and September 2023. The search strategy encompassed three databases, identifying 11 articles that met the inclusion criteria.

**Results:**

Unintended consequences were organized into four themes: disordered weight control behaviors, parental discomfort or encouragement of disordered weight control behaviors, eating disorder triggers, and financial losses. The analysis of disordered weight control behaviors indicates limited impact on youth, and we noted limited consensus in the assessment of these behaviors. We observed parent concerns about BMI screening and reporting as well as apprehensions about privacy and efficacy. There were fewer articles addressing eating disorder antecedents, although there was evidence that some youths with eating disorders considered school health class a trigger of their disorder. One study was identified that found an increase in food waste following replacement of sugar-sweetened beverages.

**Implications:**

Findings underscore the importance of comprehensive evaluation and consideration of unintended consequences in the development and implementation of school-based health policies. Recommendations include further longitudinal research, integrating obesity prevention with eating disorder prevention, and de-implementation when unintended consequences potentially outweigh benefits, such as in BMI screening and surveillance.

**Systematic Review Registration:** Identifier CRD42023467355. https://www.crd.york.ac.uk/prospero/display_record.php?RecordID=467355

## 1 Introduction

Childhood obesity and nutrition have been central foci of public health interventions for over a decade in the United States, garnering over 1.2 billion dollars of funding from the National Institutes of Health in the last 5 years alone (National Institutes of Health, 2023). Much of this attention and resources has been allotted to school-based interventions or policies. The motivation behind this rests on the assumption that public school systems are considered to transcend the limitations of socioeconomic status and, thus, can provide more equitable health interventions on a large scale (School Health Guidelines to Promote Healthy Eating and Physical Activity, n.d.). However, less attention has been given to the potential impacts of these interventions outside of their intended target of obesity reduction and prevention, otherwise known as their unintended consequences.

Unintended consequences are an important consideration in examining the efficacy of any public health research and may be particularly important for school-based obesity policies for several reasons. First, these programs can be costly and, thus, should undergo thorough testing to ensure both efficacy and cost-effectiveness in achieving program aims. Second, considering that risk for developing disordered eating and eating disorders peaks during early adolescence (Rohde et al., 2015), it is critical to ensure that interventions delivered to youth in this age group do not unintentionally contribute to symptom onset. Third, evidence indicates that overweight and obese adolescents are far more likely to develop disordered weight control behaviors (DWCBs) than those who are not overweight or obese (De Giuseppe et al., 2019). This vulnerability suggests the need for heightened awareness of unintended consequences of weight focused public health interventions. Fourth, weight-related teasing is rampant in adolescence and is associated with eating disorder onset (Lin et al., 2023), body dissatisfaction (Fields et al., 2021), and poor psychological wellbeing (Blanco et al., 2020). If obesity prevention programs imply a hierarchy of body sizes (i.e., that smaller bodies are preferable to larger ones), they may inadvertently contribute to weight stigma and existing weight-related teasing in school settings.

There is evidence to suggest that obesity prevention programs may contribute to the development of eating disorders and DWCBs. Targeted prevention programs focus on youth in larger bodies, and the primary aims of obesity prevention programs often include diet change and increases in physical activity. These behavior changes may precede the use of more maladaptive weight control behaviors, particularly for adolescents with a higher body mass index (BMI) who are already at significantly greater risk factor for disordered eating behaviors (Flament et al., 2015; Stabouli et al., 2021). Importantly, the risks for the development of eating disorders following obesity-prevention programs is likely multi-faceted and complex (Leme et al., 2020; Lin et al., 2023). Factors such as socioeconomic status, for example, may increase risk of both obesity and DWCBs (Mohammed et al., 2019; Accurso et al., 2021).

Though recommended by experts, it is not currently standard practice to evaluate unintended consequences in health policy research (Oliver et al., 2019). Understanding unintended adverse effects may be particularly important in the case of school-based obesity prevention policies. In this systematic review, we examined the existing literature on unintended consequences of school-based health initiatives and developed recommendations for future school-based policies or interventions. By understanding the unintended consequences of school-based policies, we can modify the design and/or implementation of interventions so that they support children’s wellbeing while reducing unintentional harm.

## 2 Methods

This is a systematic review of quantitative, qualitative, and mixed methods research pertaining to unintended consequences of school health and nutrition policies. We followed Preferred Reporting Items for Systematic Reviews and Meta-Analyses (PRISMA) guidelines and registered our protocol on PROSPERO systematic review registry under identification number CRD42023467355. The methods did not deviate from the review’s registration.

### 2.1 Search strategy

Studies were identified via three databases: PubMed, CINAHL, and Scopus. Searches occurred on October 5, 2023 and were limited to January 2013–September 2023. SLT conducted the search with assistance of an institutional librarian. We included the following key words in our search: school health policies; educational policy effects; education policy outcomes; school health interventions; health curricula; K-12 students; student well-being; student health outcomes; unintended consequences; adverse effects of school policies; policy evaluation; policy side effects; unanticipated outcomes; policy unintended outcomes; psychological distress; weight related teasing; DWCBs; eating disorder symptomatology; weight concerns; food insecurity; weight stigma; United States. The electronic search strategy was expanded upon with a manual search of reference lists from the final sample of articles.

#### 2.1.1 Criteria

Inclusion criteria were developed in the PICOT format and were as follows:

- Population: K-12 students, teachers, or parents/family members in the United States. Articles were restricted to the United States as school policy and funding streams vary widely between countries, thus we restricted searches to one country.
- Intervention: Investigates a school-based health or nutrition policy, program, intervention, or curriculum
- Study Characteristics: Original research
- Outcome: Measures an unintended consequence of the policy, intervention, or curriculum (such as psychological distress, bullying/weight-related teasing, DWCBs, eating disorder symptomatology, weight and shape concerns, food insecurity, or weight stigma)
- Timeline: Published between January 2013–September 2023.

Exclusion criteria included studies that occurred outside of the United States, studies that focused on other educational settings (such as college or early childhood education), and studies that investigated policies related to other aspects of health such as dental care, vision, hearing, or postural screening.

#### 2.1.2 Screening

We screened articles via the Covidence systematic review software (Veritas Health Innovation, 2024). This particular software was selected for its structured workflow (matching the requirements of a Cochrane systematic review), its enforcement of blinding during article screening and resolution of conflicts, and its ability to facilitate data-extraction. All titles and abstracts were screened for initial inclusion based on the criteria noted above by two of three independent reviewers (AL, AA, ZP). Where discrepancies occurred, SLT guided the final team decision. The same process occurred for full text screening.

### 2.2 Data extraction and synthesis

Three independent coders (AL, GH, ZP) abstracted data from included articles, with SLT guiding team consensus when extraction results differed. The following data fields were extracted from each article: Title, authors, year of publication, journal of publication, funding sources, type of intervention being studied, stated aim of study, study design, description of intervention or policy, named intention of policy or intervention, unintended consequence examined, study population, inclusion and exclusion criteria, recruitment approach, sample size, findings related to unintended consequence, stated recommendations for future research/policy/practice, stated limitations, quality assessment. After extraction, data were analyzed and synthesized by SLT and GH.

#### 2.2.1 Quality assessment

Each included article was assessed for quality using the LEGEND Evidence Evaluation guidance (LEGEND, n.d.). The LEGEND system offers guidance on quality appraisal of research with an eye toward the implementation of evidence-based practices. The LEGEND Evidence Evaluation system categorizes studies on a scale from 1 to 5, with 1 being the highest level of evidence. Each level also includes a quality ranking (a or b) where “a” indicates a good quality study and “b” indicates a lesser quality study.

## 3 Results

### 3.1 Study selection

**Figure 1:**
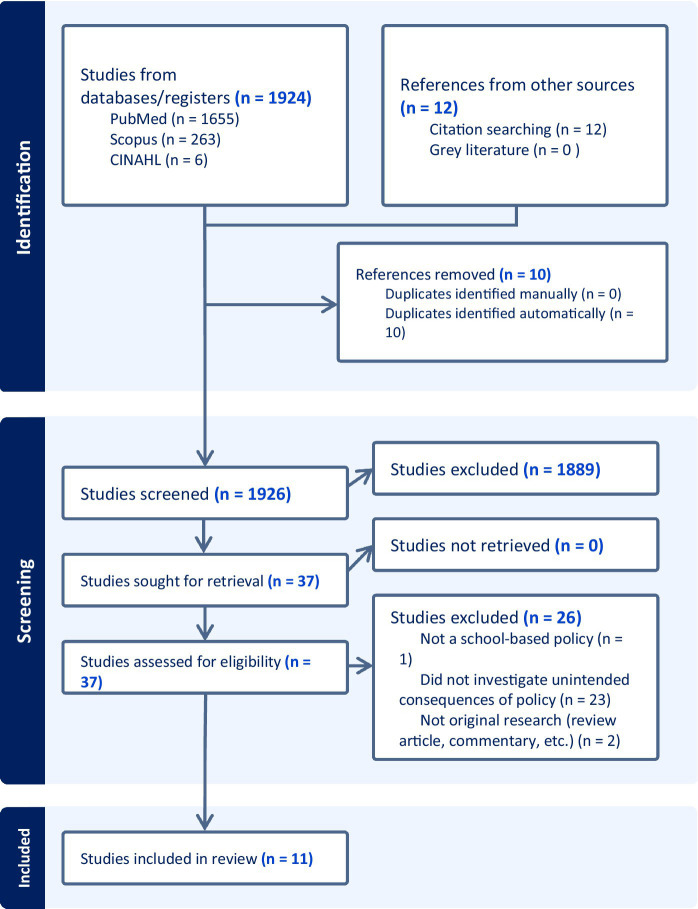
PRISMA study flow diagram.

A full depiction of the screening process is described in the PRISMA study flow diagram in Figure 1. The initial database search identified 1,924 records from the three databases searched with an additional 12 references identified via reference list searches. Our final sample comprised 11 articles for data extraction (see Table 1).

**Table 1:** Summary of included studies.

Author, Year	Type of inter-vention	Study design	Study population	Sample size	Named intention of policy or intervention	Description of intervention or policy	Unintended consequence examined	Findings related to unintended consequence	Stated recommendations for future research/policy/practice	Level of Evidence^a^
Lin et al. (2023)	School curriculum	Retrospective chart review, qualitative thematic analysis	Youth diagnosed with an eating disorder and admitted for medical treatment	150	Health education	Health education as part of standard curricula	Self-reported eating disorder triggers	14% (*n* = 9) of respondents indicated that receiving health education was an initial trigger of their eating disorder	Additional research to understand the impact of “healthy lifestyle” intervention may affect adolescent development	4a
Lee and Kubik (2015)	District-level policy	Secondary data analysis of a cross-sectional research study	Parents of students who participated in BMI screening program	122	Lifestyle change; health promotion	BMI screening and report to parents	Parental food restriction and offering of diet pills/herbal supplements after BMI screening	8.1% (*n* = 10) of parents reported putting child on a diet/restricting food after receiving BMI report	Continued implementation of BMI screening and parent notification programs; additional research into effectiveness at obesity reduction	4a
Ruggieri and Bass (2016)	State-level policy	Focus groups, qualitative analysis	Female Black/African-American-identifying parents/guardians	20	Reduce and prevent obesity	(1) BMI measurements, (2) School-based BMI screening programs, (3) BMI reporting to parents	Parent perceptions of potentially negative effects of BMI screening and “BMI report cards”	Parental concern for loss of child’s confidentiality, potential impact on self-esteem, and potential of weight-related teasing as a result of screening; parental concern for overreach by school system	Schools should emphasize follow-up care from a primary care provider when implementing BMI screening to minimize independent, unsupervised weight loss	4a
Moyer et al. (2014)	State-level policy	Cohort-style qualitative focus groups.	Parents of students who participated in BMI screening program	29	Prevent and reduce obesity	BMI screening and report to parents	Parent perceptions of potentially negative effects of BMI screening and “BMI report cards”	Greater number of participants expressed concern/negative perception of BMI screening letters than those who expressed positive regard; Nonadherence to state guidelines occurred in 5 of 29 participant cases, resulting in privacy concerns; Parents voiced feelings of denial, anger, rationalization, concern, guilt, and fear.	Additional context should be added to BMI letters to enhance parents’ understanding of findings; parent-focused correspondence about child weight status should be conducted carefully due to emotional nature	3a
Zhu and Thomas (2013)	State-level policy	Longitudinal moderation analysis	School-level data	43 states	Reduce and prevent obesity	Range of policies including (1) Improved nutrition and physical activity standards, (2) mandatory BMI screening, and (3) BMI screening report to parents	Self-reported weight control behaviors	Across multiple levels of social capital, a greater number obesity prevention/reduction policies was associated with increases in weight control behaviors. Low social capital = greater prevalence of fasting for weight control in girls (*b* = 0.123; *p* < 0.01); High social capital = greater prevalence of diet pill or laxative use in boys (*b* = 0.158; *p* < 0.01).	Further research on longitudinal associations and considering socioeconomic factors in future investigations	4a
Jones et al. (2014)	School-level intervention	Feasibility study	High school students from both”overweight” and “normal weight” groups	336	Promote weight regulation; improve weight/shape concerns	Online student-facing interactive intervention	Weight and shape concerns	Across all BMI categories, no change in weight and shape concerns pre/post intervention. Those with elevated weight and shape concerns showed decrease (low BMI: mean change −1.55 [SD 12.9]; t193 = −1.68; *p* = 0.095; high BMI: mean change −3.02 [SD 15.8]; t90 = −1.82; *p* = 0.071), but still elevated above normal range.	Integration of obesity and eating disorders prevention programs and broad, universal implementation	4a
Hanks et al. (2014)	District-level policy	“Before-after” pilot study	Oregon elementary schools	11	Reduce sugar and calorie intake	Replacement of chocolate milk with skim milk	Changes in milk selection, cost effectiveness, food waste	Significant (29.4%) increase in milk waste (p < 0.001), increases in milk cost per ounce by 10.0%.	Alternative solutions should be considered and investigated (e.g., making chocolate milk less convenient to access)	4a
Taber et al. (2014)	State-level policy	Cross-sectional secondary data analysis	Public school students in grades 9–12	8,245	Reduce sugar-sweetened beverage access and consumption	Soda taxes and legislation governing soda sales in schools	Unhealthy weight-control behaviors (skipping meals, fasting, smoking, vomiting or taking laxatives, taking diet pills/powders/liquids without a doctor’s advice)	No impact noted on unhealthy weight control behaviors. Lack of access to vending machines in schools was associated with greater daily soda consumption (AME = −4.02, *p* = 0.02) and more days eating fast food per week (AME = −0.24, *p* = 0.01).	Future studies to examine longitudinal associations and mechanisms of action underpinning greater soda intake with limited access	3a
Madsen et al. (2017)	State-level policy	Cluster randomized clinical trial	California elementary and middle school students	28,641	Reduce and prevent obesity	BMI screening and report to parents (group 1) vs. BMI screening only (group 2)	Weight-related teasing, weight-related perceptions and behaviors, weight related-conversation in home	Students who were exposed to BMI screening and reporting reported slightly less weight satisfaction (3.2% reduction from baseline at 2y, *p* = 0.001) and slightly more weight talk (2.9% increase from baseline at 1y, *p* = 0.001), but less engagement in DWCBs (5.2% reduction from baseline at 1y, *p* = 0.001). Students who considered themselves “very overweight” and received the BMI screening intervention experienced greater pressure from family to diet in 2y follow up period than those who did not receive the intervention (0.44; 95% CI, 0.06–0.82).	De-implementation of BMI screening in favor of other surveillance methods (i.e., data abstraction from medical records rather than primary data collection)	2a
Phillips et al. (2013)	State-level policy	Cross-sectional mixed methods analysis (surveys and qualitative interviews)	Arkansas public schools	113	Reduce and prevent obesity	Arkansas Act 1220; Comprensive legislation to address childhood obesity in schools	Weight-related teasing; unhealthy weight control behaviors	No change noted in adolescent concern about weight, exercising for weight control, or dieting. Weight-based teasing and diet pill use decreased over time (weight-based teasing: 12% in 2004; 6% in 2010; diet pill use: 6% in 2004; 3% in 2010).	None stated	3a
Larson et al. (2017)	Multiple levels	Longitudinal secondary data analysis	Minnesota schools enrolling adolescents in grades 9–12	42	Health promotion; obesity prevention	School-based health promotion	Self-report of healthy, unhealthy, and extreme weight control behavior	Obesity-prevention policies were unrelated to the prevalence of student weight-control behaviors	Further research examining sex-based differences in policy-related behaviors; integration of eating disorder/DWCBs behavior prevention content into current curricula or educational standards	4a

^a^Level of evidence was determined using (Clark et al., 2009) uidelines. Studies are ranked according to level of evidence on a scale of 1–5 (1 = highest level, 5 = lowest) and annotated with quality assessment a or b (*a* = high quality, *b* = lesser quality).

### 3.2 Study characteristics

#### 3.2.1 Study quality

All included studies were of acceptable quality according to LEGEND guidance, earning an “a” distinction. Most designs were categorized as level 4 evidence (*n* = 7, 63.6%), while 3 were categorized as level 3 (27.2%), and 1 was categorized as level 2 (9%).

#### 3.2.2 Study design

Methodology varied across studies. Two studies (Moyer et al., 2014; Ruggieri and Bass, 2016) utilized only qualitative methods, both employing focus groups. Of these, one (Moyer et al., 2014) utilized a longitudinal focus group approach, where focus groups were conducted multiple times throughout the implementation of a BMI screening program. Two other studies (Phillips et al., 2013; Lin et al., 2023) employed mixed methods, and the remaining seven studies (Zhu and Thomas, 2013; Hanks et al., 2014; Jones et al., 2014; Taber et al., 2014; Lee and Kubik, 2015; Larson et al., 2017; Madsen et al., 2017) used only quantitative methods. Designs of the quantitative studies were cross-sectional (*n* = 2) (Taber et al., 2014; Lee and Kubik, 2015), pre/post (*n* = 2) (Hanks et al., 2014; Jones et al., 2014), longitudinal (*n* = 2) (Zhu and Thomas, 2013; Larson et al., 2017) and one randomized clinical trial (Madsen et al., 2017).

#### 3.2.3 Sample population and size

We observed a wide range of populations and sample sizes across studies. Four studies investigated trends and phenomena among youth (Jones et al., 2014; Taber et al., 2014; Madsen et al., 2017; Lin et al., 2023). The total sample of these studies was 37,372 youth (range: 150–28,641), with 28,641 coming from one randomized clinical trial (Madsen et al., 2017). Three studies investigated parents of youth (Moyer et al., 2014; Lee and Kubik, 2015; Ruggieri and Bass, 2016), with a total sample size across studies of 171 (range: 20–122). The remaining four studies (Phillips et al., 2013; Zhu and Thomas, 2013; Hanks et al., 2014; Larson et al., 2017) analyzed school- and state-level data and included data from a combined 66 schools and 43 states.

### 3.3 Policies and interventions examined

Studies varied in terms of the breadth and scope of interventions that were investigated. Six of the included studies (Phillips et al., 2013; Zhu and Thomas, 2013; Moyer et al., 2014; Taber et al., 2014; Ruggieri and Bass, 2016; Madsen et al., 2017) examined state-level policies, three studied individual school or district-level policies or interventions (Hanks et al., 2014; Jones et al., 2014; Lee and Kubik, 2015), one investigated policies at varying levels of impact (Larson et al., 2017), and one specifically investigated the impact of school health curricula (Lin et al., 2023). All included studies framed the policy or intervention under investigation as an obesity reduction, prevention, or surveillance initiative. Two articles (Hanks et al., 2014; Taber et al., 2014) specifically noted the reduction of sugar-sweetened beverages as additional foci.

The mechanisms used to promote obesity reduction/prevention varied across studies. Unintended consequences were most commonly assessed in the context of BMI screening or surveillance (*n* = 6) (Phillips et al., 2013; Zhu and Thomas, 2013; Moyer et al., 2014; Lee and Kubik, 2015; Ruggieri and Bass, 2016; Madsen et al., 2017). Of note, school-based BMI screening was often discussed as a two-part intervention: (1) the collecting of BMI data in the school setting, and (2) the report of that data to parents and guardians. Other articles examined the impact of nutrition-focused health class curricula (Jones et al., 2014; Lin et al., 2023), limiting access to or imposing taxes on sugar-sweetened beverages (Hanks et al., 2014; Taber et al., 2014), and broadly improved school health and nutrition standards (Zhu and Thomas, 2013; Larson et al., 2017).

### 3.4 Unintended consequences

The unintended consequences examined by each study were dependent upon the intervention and population being investigated. A full description of each study and findings related to unintended consequences is available in Table 1. Of note, we excluded “dieting” as an unintended consequence, as caloric reduction or dietary changes were an explicit goal of many programs. The exception was when the authors specifically addressed dieting as an explicit unintended consequence, which was usually in the context of DWCBs. In general, unintended consequences comprised four categories: DWCBs, impact on parents, eating disorder triggers, and financial losses.

#### 3.4.1 Disordered weight control behaviors (DWCB) and weight/shape concerns among youth

Five studies examined the impact of policies or interventions on the development of DWCBs among youth (Zhu and Thomas, 2013; Taber et al., 2014; Lee and Kubik, 2015; Larson et al., 2017; Madsen et al., 2017). Although the literature has defined DWCBs as subclinical eating disorder symptoms (including weight loss dieting, binge eating, self-induced vomiting, dysfunctional exercise, and the use of laxatives or diuretics) (López-Gil et al., 2023), studies in this review used a variety of operational definitions. Larson et al. (2017) categorized behaviors into “healthy, unhealthy, and extreme” weight control behaviors. Exercising and “healthy eating” were healthy behaviors, fasting, skipping meals or smoking cigarettes to curb hunger were unhealthy behaviors, and purging (diet pills, laxative use, vomiting) and drug use (for weight control reasons) were considered extreme. Other studies relied on students to self-define weight control behaviors as unhealthy or maladaptive. The most common and seemingly agreed-upon behaviors that constituted DWCBs were fasting, purging via vomiting or laxatives, taking diet pills or appetite suppressants, and smoking for weight control.

The studies that investigated DWCBs as unintended consequences of school policies generally agreed that there was no clinically significant impact on the development of DWCBs. One exception was Zhu and Thomas (2013), who examined obesity reduction and prevention policies as they relate to social capital and DWCBs. Specifically, they found that obesity reduction programs led to increased use of DWCBs, though the specific DWCBs used by youth differed by social capital and gender. In contrast, Phillips et al. (2013) and Madsen et al. (2017). noted modest decreases in DWCBs over time, ranging from 3 to 6% over 2–6 years. One study examined the impact of a student-facing online intervention on student weight and shape concerns (Jones et al., 2014), also noting no clinically or statistically significant change in weight/shape concerns among those who received the intervention. Overall, there was little to no impact on DWCBs, indicating that obesity prevention and reduction programs may not be associated with this unintended consequence.

#### 3.4.2 Parental discomfort or parental encouragement of DWCBs

Four studies investigated the unintended impacts of policies on parents or caregivers (Moyer et al., 2014; Lee and Kubik, 2015; Ruggieri and Bass, 2016; Madsen et al., 2017). Of these, two measured parental discomfort (Moyer et al., 2014; Ruggieri and Bass, 2016), both noting loss of confidentiality and potential impacts on student self-esteem as contributors to parental discomfort. Two studies measured the impact of policies on parental encouragement of DWCBs. Lee and Kubik (Lee and Kubik, 2015) noted that 8.1% (*n* = 10) of parents reported putting their child on a diet or restricting their food after receiving BMI screening results and Madsen et al. (2017). noted that students whose families received BMI screening results experienced significantly more pressure from parents to engage in dieting behaviors at 2-year follow up compared to those who did not. This preliminary evidence suggests that obesity prevention and reduction interventions may negatively impact family systems via parent behavior change.

#### 3.4.3 Eating disorder triggers

One study retrospectively asked youth who are currently diagnosed with an eating disorder to describe the initial trigger of their eating disorder (Lin et al., 2023). Results indicated that 14% (*n* = 9) of participants perceived a school health course or curriculum was an initial trigger of their eating disorder. Though studies exploring eating disorder triggers were underrepresented in the included literature, this finding suggests that a substantial minority of participants were negatively impacted by health programs implemented in a school setting.

#### 3.4.4 Financial losses

One study examined the cost-effectiveness of replacing sugar-sweetened beverages, specifically chocolate milk (Hanks et al., 2014). Authors concluded that the removal of chocolate milk from elementary schools contributed to a significant (29.4%, *p* < 0.001) increase in milk waste, which contributed to a net increase in milk cost of 10.0%. Especially for individuals who struggle with inadequate resources, this highlights a significant area of concern – particularly when considering the implications of increasing nutrition costs and their role in food insecurity.

## 4 Discussion

In this review, we systematically investigated the unintended consequences of school-based health and nutrition policies implemented within the United States. The aims of this review were to determine if sufficient evaluation of weight-focused interventions implemented in schools has occurred, and if necessary, elucidate the unintended consequences of these interventions to inform and shape future implementation and research efforts. Main findings indicated that obesity prevention and reduction-focused policies had a marginal impact on youth themselves, but we found greater evidence for an impact on families and caregivers, related to both personal discomfort or disapproval and parental behaviors. Other important findings highlight concerns related to nutritional costs and the development of DWCBs.

Multiple studies in this review examined the impact of school-based BMI screening and the reporting of results to families (*n* = 6, 54.5%). Findings of this review are consistent with prior literature, which suggests ample concern from parents about the necessity, privacy, and efficacy of BMI screening in schools (Tatum et al., 2021). In recent years, a shift away from BMI surveillance and reporting has been recommended by experts (Egan et al., 2023) after a growing body of evidence suggested poor efficacy in the their intended goal of reducing obesity (Ikeda et al., 2006; Gee, 2015). Furthermore, school-based BMI screening does not meet the standards set by the American Academy of Pediatrics recommending specific criteria for the employment of screening tests in schools (Nihiser et al., 2009). The recommendations made included studies were contradictory regarding next steps for BMI screening. While (Lee and Kubik, 2015) recommended the continued use of BMI screening in schools, Madsen et al. (2017). recommended de-implementation. Two other investigations of BMI screening (LEGEND, n.d.; Veritas Health Innovation, 2024) recommended the utilization of safeguards when implementing BMI screening in schools, such as emphasizing the purpose of screening as connecting with a healthcare provider to discuss results or adding context to results letters shared with parents. Of note, the most recent, comprehensive expert recommendations did recommend de-implementation of BMI screening and surveillance nationwide (Zhu and Thomas, 2013). Considering the risk vs. benefit, we recommend the continued de-implementation of BMI screening and surveillance in school settings.

Much of the literature on unintended consequences of school-based BMI screenings remains parent-focused. There is a paucity of evidence examining the unintended consequences of BMI screening on youth themselves, despite being the intended targets of these interventions. A greater understanding of any unintended consequences on students could be instrumental in advocating for de-implementation at the policy level. There is similarly lacking literature examining whether the documented parental impacts actually influence youth behaviors, though it is well-established that youth whose parents engage in DWCBs are more likely to do so themselves (Erriu et al., 2020; Parenting Styles and Eating Disorder Pathology, n.d.).

Overall, our findings suggest that obesity prevention and reduction policies did not substantially increase DWCBs in youth. However, we also overarchingly saw no, or marginal improvement, in DWCBs following interventions. Though the role of DWCBs and eating disorder prevention in relation to obesity prevention is heavily debated, our results suggest that obesity prevention may not significantly impact the prevalence of these behaviors in *some samples*. Rather than preventing or avoiding DWCBs, our results may point to the limited impact of anti-obesity interventions writ large (Ikeda et al., 2006; Gee, 2015; Liu et al., 2019). Indeed, a recent meta-analysis indicated that although anti-obesity programs generally showed some reduction in BMI compared to those who did not receive the intervention (Liu et al., 2019), the reduction in BMI was not clinically significant (Reinehr et al., 2016). Thus, it is likely that these interventions have limited impact. It should also be noted that since it is not a named intention of the policies examined, the assessment of DWCBs is often a secondary aim of the research, and thus may not be conducted as rigorously as possible. Further investigation that more rigorously assesses the prevalence of DWCBs, the change in rate of DWCBs within different weight groups (such as youth with overweight and obesity), and weight or shape concerns is warranted.

A consistent theme emerging from the analysis of these data was variable and often unvalidated measurement of “disordered” or “unhealthy” weight control behavior. Though some experts posited definitions of the term (Leal et al., 2020), authors of articles included in this review largely defined the term ideographically. For the purposes of this review, dieting was explicitly excluded. Though substantial evidence suggests dieting behavior in childhood is maladaptive and contributes to the development of eating disorders (Elran-Barak and Bar-Anan, 1982; Rohde et al., 2015; Leme et al., 2020; Stabouli et al., 2021), so-called “dieting,” or limiting certain food groups in favor of lower-calorie or lower-fat options, is often the *intention* of obesity reduction policies. Thus, it is difficult to determine whether increases in dieting behavior are truly *unintended* consequences of these policies. However, caloric restriction can be harmful – especially during a period of significant physical, social, and psychological development such as adolescence. There is no way to determine when or if the reduction in calories reported in included articles went beyond what was safe/recommended in each intervention. Therefore, it is possible that the prevalence of DWCB is underestimated as the degree of caloric restriction and any subsequent negative impact on the body (i.e., amenorrhea, orthostasis) is typically not tracked or reported. When examining unintended consequences, researchers should clarify their definition of “dieting” and should consider addressing it in more nuanced way.

Recommendations and implications differed widely in scope and impact among included articles. The most frequent recommendation was to conduct further research, especially more deeply into unintended consequences of policies, as existing literature is limited (Hanks et al., 2014; Jones et al., 2014; Lee and Kubik, 2015; De Giuseppe et al., 2019). Specifically, authors called for a focus on longitudinal investigations and further inquiry into sex-based differences in intervention response. Jones et al. (2014). recommended the integration and study of obesity prevention alongside eating disorder prevention, which has been debated in the research as many obesity prevention programs focus on decreasing caloric intake and limiting certain food groups, which are known risk factors for eating disorders (Flament et al., 2015; Blanco et al., 2020). However, results of this review did not suggest evidence of this link materializing into DWCBs or eating disorders.

Eating disorder triggers and financial losses also emerged as themes of concern, although they were addressed less frequently. Lin et al. concluded that a sizeable portion (14%, *n* = 9) of their sample attributed the development of their eating disorder to their school health class content (Lin et al., 2023). It is important to note that eating disorders have a multifaceted etiology that is not yet fully understood, and that school health class curricula are likely not solely responsible for the development of an eating disorder (Weissman, 2019). However, the fact that many youth perceived their health class as causing enough harm to contribute to their disease warrants alarm, and thus, experts designing curricula should consult with experts in eating disorder development to ensure minimal potential for harm.

### 4.1 Strengths and limitations

This review is a novel examination of the unintended consequences of school health policies in response to calls for the examination of unintended consequences as a foci of policy implementation research (Oliver et al., 2019). We conducted a comprehensive review of literature from multiple far-reaching databases and utilized dynamic search terms to ensure the capture of as much relevant literature as possible. However, our review does have limitations to be considered. First, we elected to include studies that employed various methodologies (quantitative, qualitative, and mixed methods), which made the comparison of included articles more difficult and complex. We ultimately elected to do so as the nature of unintended consequences is such that they were not premeditated and can easily be overlooked, and thus we wanted to be as comprehensive as possible in our evaluation of them. Because of these differing methodologies, we were unable to conduct a meta-analysis of data.

Although we made a significant effort to be exhaustive in our searches, it is possible that relevant articles were not identified and not included in this review. It is also possible that some unintended consequences may have been inadvertently excluded by our search strategy. To narrow the search strategy and scope of this review, there was an assumed negativity to the unintended consequences included. We acknowledge that some unintended consequences of policy do in fact have a positive impact, and thus, future literature and systematic reviews should investigate positive consequences.

## 5 Conclusion

School-based health and nutrition policies are common throughout the United States; however, investigation into their unintended consequences is minimal. Findings of this review indicate the need for more longitudinal research to better examine unintended consequences. Unintended consequences should be considered throughout all phases of policy and intervention development, including post-deployment. As DWCBs are the most common unintended consequence considered, it is important that we accurately assess these behaviors – specifically regarding the intended behavior changes of these policies. A key element to understanding the impact of unintended consequences is interpreting the consequences in the context of the intervention’s effectiveness; when the unintended consequences outweigh the benefits of the intervention, de-implementation is warranted.

## Data availability statement

No original datasets were generated or analyzed during the current study. All abstracted data is summarized in the text. Data exported from Covidence is available upon request from the the corresponding authors.

## Author contributions

SLT: Writing – original draft, Methodology, Formal analysis, Data curation, Conceptualization. AML: Writing – review & editing, Investigation. GH: Writing – original draft, Investigation, Formal analysis. ZP: Writing – review & editing, Investigation. AA: Writing – review & editing, Investigation. CAT: Writing – review & editing, Conceptualization.

## References

[ref1] Accurso E. C. Buckelew S. M. Snowden L. R. (2021). Youth insured by Medicaid with restrictive eating disorders—Underrecognized and Underresourced. JAMA Pediatr. 175, 999–1000. doi: 10.1001/jamapediatrics.2021.2081, PMID: 34338735 PMC8896396

[ref2] Blanco M. Solano S. Alcántara A. I. Parks M. Román F. J. Sepúlveda A. R. (2020). Psychological well-being and weight-related teasing in childhood obesity: a case-control study. Eat Weight Disord. EWD 25, 751–759. doi: 10.1007/s40519-019-00683-y, PMID: 31077019

[ref036] Clark E. Burkett K. Stanko-Lopp D. (2009). Let Evidence Guide Every New Decision (LEGEND): An evidence evaluation system for point-of-care clinicians and guideline development teams. Journal of Evaluation in Clinical Practice 15, 1054–1060. doi: 10.1111/j.1365-2753.2009.01314.x 20367705

[ref3] De Giuseppe R. Di Napoli I. Porri D. Cena H. (2019). Pediatric obesity and eating disorders symptoms: the role of the multidisciplinary treatment. A systematic review. Front. Pediatr. 7:123. doi: 10.3389/fped.2019.00123, PMID: 31024868 PMC6463004

[ref4] Egan N. Cory H. Goldberg D. S. Gordon A. Jordan J. Kavanaugh J. R. . *Report: De-Implementation of BMI Surveillance*. (2023). https://www.hsph.harvard.edu/striped/wp-content/uploads/sites/1267/2023/09/STRIPED-Report-De-Implementation-of-BMI-Surveillance.pdf (Accessed December 14, 2023).

[ref5] Elran-Barak R. Bar-Anan Y. (1982). Implicit and explicit anti-fat bias: the role of weight-related attitudes and beliefs. Soc. Sci. Med. 204, 117–124. doi: 10.1016/j.socscimed.2018.03.018, PMID: 29655062

[ref6] Erriu M. Cimino S. Cerniglia L. (2020). The role of family relationships in eating disorders in adolescents: a narrative review. Behav. Sci. 10:71. doi: 10.3390/bs10040071, PMID: 32252365 PMC7226005

[ref7] Fields L. C. Brown C. Skelton J. A. Cain K. S. Cohen G. M. (2021). Internalized weight Bias, teasing, and self-esteem in children with overweight or obesity. Child Obes. Print. 17, 43–50. doi: 10.1089/chi.2020.0150, PMID: 33351706 PMC7815063

[ref8] Flament M. F. Henderson K. Buchholz A. Obeid N. Nguyen H. N. T. Birmingham M. . (2015). Weight status and DSM-5 diagnoses of eating disorders in adolescents from the community. J. Am. Acad. Child Adolesc. Psychiatry 54, 403–411.e2. doi: 10.1016/j.jaac.2015.01.020, PMID: 25901777

[ref9] Gee K. A. (2015). School-based body mass index screening and parental notification in late adolescence: evidence from Arkansas’s act 1220. J. Adolesc. Health 57, 270–276. doi: 10.1016/j.jadohealth.2015.05.007, PMID: 26115907

[ref10] Hanks A. S. Just D. R. Wansink B. (2014). Chocolate Milk consequences: a pilot study evaluating the consequences of banning chocolate Milk in school cafeterias. PLoS One 9:e91022. doi: 10.1371/journal.pone.0091022 24740451 PMC3989166

[ref11] Ikeda J. P. Crawford P. B. Woodward-Lopez G. (2006). BMI screening in schools: helpful or harmful. Health Educ. Res. 21, 761–769. doi: 10.1093/her/cyl144 17093140

[ref12] Jones M. Taylor Lynch K. Kass A. E. Burrows A. Williams J. Wilfley D. E. . (2014). Healthy weight regulation and eating disorder prevention in high school students: a universal and targeted web-based intervention. J. Med. Internet Res. 16:e57. doi: 10.2196/jmir.2995, PMID: 24583683 PMC3962843

[ref13] Larson N. Davey C. S. Caspi C. E. Kubik M. Y. Nanney M. S. (2017). School-based obesity-prevention policies and practices and weight-control behaviors among adolescents. J. Acad. Nutr. Diet. 117, 204–213. doi: 10.1016/j.jand.2016.09.030, PMID: 27889315 PMC5276726

[ref14] Lee J. Kubik M. Y. (2015). Child’s weight status and Parent’s response to a school-based body mass index screening and parent notification program. J. Sch. Nurs. 31, 300–305. doi: 10.1177/1059840514556181, PMID: 25377929

[ref15] LEGEND. Evidence Evaluation Tools & Resources | James M. Anderson Center for Health Systems Excellence. Accessed December 14, 2023. Available at: https://www.cincinnatichildrens.org/research/divisions/j/anderson-center/evidence-based-care/legend

[ref16] Leme A. C. B. Haines J. Tang L. Dunker K. L. L. Philippi S. T. Fisberg M. . (2020). Impact of strategies for preventing obesity and risk factors for eating disorders among adolescents: a systematic review. Nutrients 12:3134. doi: 10.3390/nu12103134, PMID: 33066501 PMC7602154

[ref17] Lin J. A. Jhe G. Adhikari R. Vitagliano J. A. Rose K. L. Freizinger M. . (2023). Triggers for eating disorder onset in youth with anorexia nervosa across the weight spectrum. Eat. Disord. 31, 553–572. doi: 10.1080/10640266.2023.2201988, PMID: 37039575

[ref18] Liu Z. Xu H. M. Wen L. M. Peng Y. Z. Lin L. Z. Zhou S. . (2019). A systematic review and meta-analysis of the overall effects of school-based obesity prevention interventions and effect differences by intervention components. Int. J. Behav. Nutr. Phys. Act. 16:95. doi: 10.1186/s12966-019-0848-8, PMID: 31665040 PMC6819386

[ref19] López-Gil J. F. García-Hermoso A. Smith L. Firth J. Trott M. Mesas A. E. . (2023). Global proportion of disordered eating in children and adolescents: a systematic review and Meta-analysis. JAMA Pediatr. 177, 363–372. doi: 10.1001/jamapediatrics.2022.5848, PMID: 36806880 PMC9941974

[ref20] Madsen K. A. Linchey J. Ritchie L. Thompson H. R. (2017). The fit study: design and rationale for a cluster randomized trial of school-based BMI screening and reporting. Contemp. Clin. Trials 58, 40–46. doi: 10.1016/j.cct.2017.05.005, PMID: 28479218 PMC6010055

[ref21] Mohammed S. H. Habtewold T. D. Birhanu M. M. Sissay T. A. Tegegne B. S. Abuzerr S. . (2019). Neighbourhood socioeconomic status and overweight/obesity: a systematic review and meta-analysis of epidemiological studies. BMJ Open 9:e028238. doi: 10.1136/bmjopen-2018-028238, PMID: 31727643 PMC6886990

[ref22] Moyer L. J. Carbone E. T. Anliker J. A. Goff S. L. (2014). The Massachusetts BMI letter: a qualitative study of responses from parents of obese children. Patient Educ. Couns. 94, 210–217. doi: 10.1016/j.pec.2013.10.016, PMID: 24290240 PMC4553945

[ref23] National Institutes of Health. Report. (2023). https://report.nih.gov/funding/categorical-spending#/ (Accessed November 28, 2023).

[ref24] Nihiser A. J. Lee S. M. Wechsler H. McKenna M. Odom E. Reinold C. . (2009). BMI measurement in schools. Pediatrics 124, S89–S97. doi: 10.1542/peds.2008-3586L 19720672

[ref25] Oliver K. Lorenc T. Tinkler J. Bonell C. (2019). Understanding the unintended consequences of public health policies: the views of policymakers and evaluators. BMC Public Health 19:1057. doi: 10.1186/s12889-019-7389-6, PMID: 31387560 PMC6685223

[ref26] Parenting Styles and Eating Disorder Pathology. ScienceDirect. Available at: https://www-sciencedirect-com.proxy.library.upenn.edu/science/article/pii/S0195666309000385?casa_token=-RRWlzVBgw8AAAAA:TkR8XcyJW8k_qZjlH_5mT62In1IWTN6o1sZ7fhMsm5MV7i-ZyPTo6K292H-lcaeQMSzkr_kBmPAAsQ (Accessed December 14, 2023).

[ref27] Leal G. V. D. S. Philippi S. T. Dos S A. M. (2020). Unhealthy weight control behaviors, disordered eating, and body image dissatisfaction in adolescents from São Paulo, Brazil. Braz. J. Psychiatry 42, 264–270. doi: 10.1590/1516-4446-2019-0437, PMID: 32022158 PMC7236168

[ref28] Phillips M. M. Raczynski J. M. West D. S. Pulley L. Bursac Z. Leviton L. C. (2013). The evaluation of Arkansas act 1220 of 2003 to reduce childhood obesity: conceptualization, design, and special challenges. Am. J. Community Psychol. 51, 289–298. doi: 10.1007/s10464-012-9538-2, PMID: 22739790

[ref29] Reinehr T. Lass N. Toschke C. Rothermel J. Lanzinger S. Holl R. W. (2016). Which amount of BMI-SDS reduction is necessary to improve cardiovascular risk factors in overweight children? J. Clin. Endocrinol. Metab. 101, 3171–3179. doi: 10.1210/jc.2016-1885, PMID: 27285295

[ref30] Rohde P. Stice E. Marti C. N. (2015). Development and predictive effects of eating disorder risk factors during adolescence: implications for prevention efforts. Int. J. Eat. Disord. 48, 187–198. doi: 10.1002/eat.22270, PMID: 24599841 PMC4156929

[ref31] Ruggieri D. G. Bass S. B. (2016). African-American parents’ knowledge and perceptions about BMI measurements, school-based BMI screening programs, and BMI report cards: results from a qualitative investigation and implications for school-to-parent communication. J. Racial Ethn. Health Disparities 3, 320–330. doi: 10.1007/s40615-015-0149-0, PMID: 27271073

[ref32] School Health Guidelines to Promote Healthy Eating and Physical Activity. (2011) Centers for Disease Control and Prevention, Division of Population Health, National Center for Chronic Disease Prevention and Health Promotion. School health guidelines. Available at: https://www.cdc.gov/healthyschools/npao/strategies.htm

[ref33] Stabouli S. Erdine S. Suurorg L. Jankauskienė A. Lurbe E. (2021). Obesity and eating disorders in children and adolescents: the bidirectional link. Nutrients 13:4321. doi: 10.3390/nu13124321, PMID: 34959873 PMC8705700

[ref34] Taber D. R. Chriqui J. F. Vuillaume R. Chaloupka F. J. (2014). How state taxes and policies targeting soda consumption modify the association between school vending machines and student dietary behaviors: a cross-sectional analysis. PLoS One 9:e98249. doi: 10.1371/journal.pone.0098249 25083906 PMC4118851

[ref35] Tatum K. L. Valenzuela J. M. Amirniroumand R. A. Brochu P. M. (2021). Parents’ perceptions of and responses to school-based body mass index screening programs—a systematic review. J. Sch. Health 91, 331–344. doi: 10.1111/josh.13003, PMID: 33655546

[ref36] Veritas Health Innovation. (2024). Covidence systematic review software, Veritas Health Innovation, Melbourne, Australia. Available at: www.covidence.org.

[ref37] Weissman R. S. (2019). The role of sociocultural factors in the etiology of eating disorders. Psychiatr. Clin. 42, 121–144. doi: 10.1016/j.psc.2018.10.009, PMID: 30704634

[ref38] Zhu L. Thomas B. (2013). School-based obesity policy, social capital, and gender differences in weight control behaviors. Am. J. Public Health 103, 1067–1073. doi: 10.2105/AJPH.2012.301033, PMID: 23597368 PMC3698728

